# Histidine buffered media maintains pH stabile during cooled transportation of human ovarian tissue

**DOI:** 10.1186/s13048-021-00861-6

**Published:** 2021-09-03

**Authors:** Susanne Elisabeth Pors, Stine Gry Kristensen, Dmitry Nikiforov, Linn Salto Mamsen, Jesus Cadenas, Vinnie Hornshøj Greve, Margit Dueholm, Claus Yding Andersen

**Affiliations:** 1 Laboratory of Reproductive Biology, The Juliane Marie Centre for Women, Children and Reproduction, Copenhagen University Hospital and Faculty of Health and Medical Sciences, University of Copenhagen, 2100 Copenhagen, Denmark; 2 The Fertility Clinic, Aarhus University Hospital, Skejby, 8200 Aarhus, Denmark

**Keywords:** Ovarian tissue cryopreservation, Transport, In vitro maturation

## Abstract

The aim of this study was to investigate whether pH is stable when transporting ovarian tissue in media buffered with either HEPES or histidine. Furthermore, if the choice of transport media impacts the in vitro maturation rate of oocytes collected in connection with ovarian tissue cryopreservation. Human ovaries (n = 34) collected for ovarian tissue cryopreservation were transported immersed in either 30 ml of HEPES buffered (follicle flushing media (Origio; Denmark)) or histidine buffered media (Custodiol®-HTK, Koehler-Chemie, Germany). Tissue was transported on ice for 4–5 h. At arrival, the ovary was weighed, and the pH of the media was measured at 0 °C. From 15 patients, immature oocytes were collected for in vitro maturation, oocytes that matured to metaphase II were evaluated. The pH measured in the HEPES buffered media (pH = 7.5 ± 0.13, n = 18) was significantly higher (*p* < 0.001) than the pH measured in the histidine buffered media (pH = 7.2 ± 0.05, n = 16). The standard deviation of pH measurements for the histidine buffered media was significantly lower than for the HEPES buffered media measurements (*p* < 0.0001). A total of 170 and 247 immature oocytes were collected and in vitro matured from ovaries transported in HEPES and histidine buffered media, respectively. The maturation rate of immature oocytes after IVM was similar in the two groups. The results show that pH in the histidine buffered media is closer to the physiological level and more stable than in HEPES buffered medium and support the use of histidine buffered media for cooled transportation of human ovaries.

## Introduction

Network-based fertility preservation programs with centralized cryobanks for ovarian tissue cryopreservation (OTC) secure a high-quality service of a highly specialized procedure. In this setting, ovarian tissue will be removed at the local hospital and transported to a central laboratory where cryopreservation is performed and storage is available. This provides patients who may be very ill with an option of having their fertility preserved with a minimal burden [4]. Centralization of OTC has been implemented in both smaller and larger countries, such as the Danish fertility preservation program and the German FertiPROTEKT network and requires cooled transportation of the ovarian tissue for 5–22 h [2, 6]. To date, the reproductive outcomes in both the Danish and the German cohorts support the feasibility of transportation and prolonged cooling of the ovarian tissue [2, 6]. To ensure optimal preservation of the ovarian tissue during transport the tissue is immersed in cold buffered media and transported on ice or in a cooling box (2–8 °C) to lower the metabolic rate of the cells and prevent tissue damage. A recent study on bovine ovarian tissue fragments reported high lactate release in the media after 24 h of ovarian tissue storage at 4 °C, indicating that the metabolic activity in the transported tissue influences the acid–base balance in the media [9]. Further, the buffer capacity of different media is influenced by temperature [1] and it is currently unknown how stable the pH is during cooled transportation of human ovarian tissue. The aim of this study was to evaluate the buffer capacity of HEPES and histidine buffered media during cooled transportation of ovarian tissue for up to 5 h in a clinical setting, and subsequently evaluate a potential impact of the buffered media on the maturation rate of immature oocytes collected and cultured in connection with OTC.

## Materials and methods

### Study population

The study population included a total of 34 women aged 14 to 40 years undergoing OTC from October 2018 to July 2020. The diagnosis of the patients included breast cancer (n = 16), hematological cancer (n = 7), gastrointestinal cancer (n = 2), brain tumor (n = 2), gestational trophoblastic disease (n = 3), and other malignant diseases (n = 4).

### Experimental setup

One ovary was laparoscopically removed at Department of Obstetrics and Gynecology, Aarhus University Hospital, and immersed in either 30 ml of HEPES buffered follicle flushing media (Origio; Denmark) or 30 ml of histidine buffered Custodiol®-HTK (Koehler-Chemie, Germany) in a 50 ml test tube. The test tube with the ovary was then placed in a Styrofoam box with crushed ice and transported by car for 4–5 h by a local transport company directly to the Laboratory of Reproductive Biology, Rigshospitalet, in Copenhagen. After arrival, the ovary was removed from the transport media, weighed on a scale, and cryopreserved according to standard practice. The transport media was placed on ice, and pH was measured using a pH meter (Fisherbrand™ Accumet™ AE150 Benchtop pH Meter, ThermoFisher), which was calibrated before each measurement. A sub-group of patients (n = 15) had consented to donate the surplus ovarian material for research. In these patients immature oocytes were collected in connection with OTC and in vitro matured (IVM) according to Nikiforov et al. [7]. The size of cumulus cell mass surrounding the oocyte (COC) was categorized in three groups and the diameter measured [7]. After 44–48 h, the number of metaphase II oocytes was evaluated.

### Statistics

Statistical analysis was done in R (version 3.4.4) by using a linear mixed-effects model with pH as the outcome and ovarian volume and transport media as explanatory variables. The frequency of maturation for immature oocyte to MII was modeled as a logistic regression mixed-effects model with maturation to MII (yes/no) as outcome and transport medium and the size of cumulus oocyte complex (0/1/2) and oocyte diameter as explanatory variables. The differences between standard deviation of the pH measurements were evaluated using a F-test.

## Results

### pH measurements

A total of 18 and 16 ovaries was transported in HEPES or histidine buffered media, respectively. The volume of the ovaries was not significantly different between the two groups (Table 1). The pH measured in the HEPES buffered media (pH = 7.5 ± 0.13) was significantly higher than the pH measured in the histidine buffered media (pH = 7.2 ± 0.05) (Table 1). Furthermore, the standard deviation of pH measurements for the histidine buffered media was significantly lower than for the HEPES buffered media measurements (*p* < 0.0001).

**Table 1:** Transport of human ovaries for cryopreservation in two types of media. Volume of ovaries and pH of media. Number of oocytes found in connection with ovarian tissue cryopreservation and in vitro maturation results

	**Transport media**		***p*-values**
	**Histidine buffered**	**HEPES buffered**	
Patients (n)	16	18	
Age/years (mean ± SD)	27.3 ± 5.9	29.0 ± 5.9	0.4
Ovarian volume/ml (mean ± SD)	7.0 ± 2.7	6.9 ± 2.8	0.6
pH (mean ± SD)	7.2 ± 0.05	7.5 ± 0.13	Mean: < 0.001 SD: *p* < 0.0001
*Results from *in vitro* maturation*			
Patients (n)	7	8	
Age/years (mean ± SD)	25.8 ± 5.9	29.4 ± 6.1	0.3
Ovarian volume/ml (mean ± SD)	7.4 ± 2.7	6.8 ± 3.2	0.7
pH (mean ± SD)	7.2 ± 0.05	7.5 ± 0.10	Mean: < 0.001 SD: *p* < 0.001
Total oocytes (n)	247	170	
MII after 48 h (n)	67	43	0.7

### Maturation rates for immature oocytes

A total of 170 and 247 immature oocytes was collected and in vitro matured from ovaries transported in HEPES and histidine buffered media, respectively. The maturation rate of immature oocytes after IVM was similar in the two groups (Table 1).

## Discussion

It is well-known from experience with transporting donor organs for transplantation that the choice of transport media is important [5], and the golden standard procedure involves storing the organ in special preservation fluid and cooled transportation at near to zero ^o^C. Most importantly, a pH around the physiological level (pH 7.3) should be maintained during transportation to limit tissue damage [1]. Our findings showed that the histidine buffered media Custodiol®-HTK was a better choice for maintaining a stable pH during transportation of human ovarian tissue on ice compared to a HEPES buffered media. These results are supported by previous studies reporting that the buffer capacity of HEPES was reduced at temperatures just above zero, whereas the buffer capacity of histidine has been shown to be stable under cold storage [1]. Moreover, current results are in line with the clinical application of the tested media. Custodiol® HTK has been developed as a preservation solution for cold organ transplantation to prolong the ischemia tolerance of the tissue [10], whereas HEPES buffered follicle flushing media has been optimized for an IVF-setting working at warm temperatures. To our knowledge histidine buffered media is not used in traditional assisted reproduction and the only experience with reproductive tissues exists from the FertiProtekt network showing reassuring reproductive outcomes [6].

In a recent study, our group demonstrated that cooled transportation of the ovary in HEPES buffered media significantly lowered the maturation rate of the immature oocytes compared to immature oocytes collected locally, without cooled transport [7]. Even though a significantly higher pH was observed in the HEPES buffed media in the current study, results showed that it did not significantly impact the maturation rate of the immature oocytes. Thus, it appears that the reduced maturation rate following transportation was caused by the cooling rather than an elevated pH [7]. This corroborates with the findings of the IVM rates being significantly reduced for oocytes transported in follicular fluid [8]. The study by Liebenthron et al., [6] showed that prolonged cold transport has no long term detrimental impact on the function of the human ovarian tissue after transplantation, however, studies in mice have found a decreased rate of implantation after transplantation of ovarian tissue stored cold for 24 h [3]. Thus, current findings should be supported by further analysis evaluating tissue function and follicle development after transport in the different buffer media.

## Conclusion

Custodiol®-HTK is currently used for overnight transportation of ovarian tissue within the German FertiPROTEKT network [6], and current results support the use of this histidine buffered media for cooled transportation of human ovaries.

## Data Availability

The datasets used and/or analysed during the current study are available from the corresponding author on reasonable request.
